# “Red-Green” or “Brown-Green” Dichromats? The Accuracy of Dichromat Basic Color Terms Metacognition Supports Denomination Change

**DOI:** 10.3389/fpsyg.2021.624792

**Published:** 2021-03-04

**Authors:** Humberto Moreira, Julio Lillo, Leticia Álvaro

**Affiliations:** ^1^Departamento de Psicología Social, del Trabajo y Diferencial, Facultad de Psicología, Universidad Complutense de Madrid, Madrid, Spain; ^2^División de Psicología, C. E. S. Cardenal Cisneros, Madrid, Spain; ^3^Departamento de Psicología Experimental, Procesos Cognitivos y Logopedia, Facultad de Psicología, Universidad Complutense de Madrid, Madrid, Spain

**Keywords:** color vision deficiencies, red-green dichromats, basic color categories, basic color terms, metacognition, color categorization, color perception, individual differences

## Abstract

Two experiments compared “Red-Green” (R-G) dichromats’ empirical and metacognized capacities to discriminate basic color categories (BCCs) and to use the corresponding basic color terms (BCTs). A first experiment used a 102-related-colors set for a pointing task to identify all the stimuli that could be named with each BCT by each R-G dichromat type (8 protanopes and 9 deuteranopes). In a second experiment, a group of R-G dichromats (15 protanopes and 16 deuteranopes) estimated their difficulty discriminating BCCs-BCTs in a verbal task. The strong coincidences between the results derived from the pointing and the verbal tasks indicated that R-G dichromats have very accurate metacognition about their capacities (they only had considerable difficulty discriminating 13 out of the total of 55 possible BCT pairs) and limitations (Brown-Green and Blue-Purple pairs were rated especially difficult to differentiate) in the use of BCTs. Multidimensional scaling (MDS) solutions derived from both tasks were very similar: BCTs in R-G dichromats were properly represented in 2D MDS solutions that clearly show one chromatic dimension and one achromatic dimension. Important concordances were found between protanopes and deuteranopes. None of these dichromats showed substantial difficulty discriminating the Red-Green pair. So, to name them “R-G” dichromats is misleading considering their empirical capacities and their metacognition. Further reasons to propose the use of the alternative denomination “Brown-Green” dichromats are also discussed. We found some relevant differences between the “Brown-Green” dichromats’ empirical and self-reported difficulties using BCTs. Their metacognition can be considered a “caricature” of their practical difficulties. This caricature omits some difficulties including their problems differentiating “white” and “black” from other BCTs, while they overestimate their limitations in differentiating the most difficult pairs (Brown-Green and Blue-Purple). Individual differences scaling (INDSCAL) analyses indicated that the metacognition regarding the use of BCTs in “Brown-Green” dichromats, especially deuteranopes, is driven slightly more by the chromatic dimension and driven slightly less by the achromatic dimension, than their practical use of BCTs. We discuss the relevance of our results in the framework of the debate between the linguistic relativity hypothesis (LRH) and the universal evolution (UE) theories.

## Introduction

The number of colors discernible by normal trichromats has been estimated to be more than 2 million (Pointer and Attridge, 1998; Martinez-Verdu et al., 2007; Linhares et al., 2008a; Kuehni, 2016). This huge number is clustered in an impressively small number of categories, which varies across languages. Languages used in technologically developed countries contain 11 (Berlin and Kay, 1969; Lin et al., 2001; Lindsey and Brown, 2014; Uusküla and Bimler, 2016; Lillo et al., 2018) or even 12 categories (Androulaki et al., 2006; Paramei, 2007; Paggetti et al., 2016; Bimler and Uusküla, 2017; Kuriki et al., 2017) while languages spoken in pre-technological cultures contain fewer categories (MacLaury, 1997; Kay et al., 2009; Brown et al., 2016). Nevertheless, this is a great reduction compared to the 2 million discernible colors. This reduction means that thousands of colors differing in their perceptual attributes (i.e., hue, saturation, and/or lightness) can belong to a single color category and, consequently, be denoted by the same term. Such a term is considered a basic color term (BCT) which denominates a basic color category (BCC) when it is used consistently among most speakers of a language (Berlin and Kay, 1969; Crawford, 1982; Corbett and Davies, 1997; Hardin and Maffi, 1997). For example, the term “red” is one of the 11 English BCTs because it allows the consistent naming of some colors sharing some perceptual characteristics (which belong to one of the 11 English BCC, red).

Interlingual differences in the number and perceptual characteristics of BCCs arise from socio-cultural differences in the need to discuss meaningful properties of object surfaces [linguistic relativity hypothesis (LRH); Saunders and van Brakel, 1997; Davidoff et al., 1999; Roberson et al., 2000; Davidoff, 2015]. On the other hand, this interlingual diversity is accompanied by interlingual similarities in BCCs focal colors and boundaries (Boynton and Olson, 1987; Lillo et al., 2007; Kay, 2015). This interlingual uniformity indicates that some universal factors related to color perception also influence the origin and evolution of BCTs, resulting in different languages including very similar BCCs [the model of Universals and Evolution (UE), Berlin and Kay, 1969; Kay and Maffi, 1999; Kay et al., 2009]. For example, the colorimetric analysis performed by Lillo et al. (2007, see also Lillo et al., 2018) showed that the American English (Boynton and Olson, 1987) and the British English (Sturges and Whitfield, 1995) include the same 11 BCCs as the Castilian Spanish. Meaning a specific English BCT (e.g., “red”) and a specific Spanish BCT (i.e., *rojo*) denote the same group of colors (the same BCC, red): both terms apply to the same set of colors, and these terms share some perceptual characteristics (specific hue, saturation and lightness ranges). Most importantly for our research: some of these characteristics are missing or distorted for people with defective color vision.

Individuals with severe forms of color vision deficiency (dichromacy) can only distinguish 7% (Linhares et al., 2008b) of the 2 million colors distinguished by normal trichromats (Pointer and Attridge, 1998; Linhares et al., 2008a; Kuehni, 2016). Consequently, dichromats are socially pressed to use the BCTs included in their language while using different perceptual referents due to their color vision deficiency. Normal trichromats have three types of cone photoreceptors in the retina: L, M, and S cones (most sensitive to long, medium, or short wavelengths, respectively). Dichromats have one fewer cone-type than trichromats as a consequence of genetic factors (Neitz and Neitz, 2011). As a result, if trichromatic cone responses to a pair of stimuli differ only in the activity of the missed cone-type, the dichromatic cone responses will be identical. Such stimuli pairs are discriminable by trichromats (different colors are perceived) but not by dichromats (the same color is perceived). Using the classical clinical nomenclature (e.g., Fletcher and Voke, 1985; Birch, 2001; Lillo et al., 2017), these stimuli are named “pseudoisochromatic.” The most common forms of dichromacy are protanopia (lack of L cones) and deuteranopia (lack of M cones). In normal trichromats, cone responses are the input signals for two chromatic opponent mechanisms, red-green and yellow-blue (Hurvich and Jameson, 1957; Hurvich, 1981). Traditionally (Hurvich and Jameson, 1955), it has been considered that protanopes and deuteranopes lack functionality in the red-green mechanism because it is based on the comparison of L and M cone responses, and one of those cone types is affected. Thus, such observers are called “red-green (R-G) dichromats.”

Considering the existence of pseudoisochromatic stimuli and assuming the lack of functionality in the red-green mechanism both in protanopes and in deuteranopes, we can easily explain how R-G dichromats respond to the color vision diagnostic tests. For example, the classical Nagel anomaloscope (see, for example, Birch, 2001) provides two hemifields with each showing different stimuli, to perform the so-called Rayleigh match. The test hemifield provides a mixture of two monochromatic lights. When presented alone, they appear as green (546 nm) and red (670 nm) for normal trichromats, because of the relative responses they produce in the L and M cones. When mixed in the adequate proportion these two lights produce the same yellowish/orangish hue than the monochromatic light (i.e., 589 nm) presented in the reference hemifield. By adjusting the intensity of the reference light, normal trichromats can achieve a perfect match (the same hue, saturation and lightness) between both hemifields. For R-G dichromats, the three monochromatic stimuli used by the Nagel anomaloscope (i.e., 546, 670, and 589 nm) only activate a cone type (M in protanopes and L in deuteranopes). It means that some stimuli that normal trichromats see as very different hues (reddish, orangish, yellowish or greenish) will appear the same hue only differing in brightness for R-G dichromats. Because of this similarity, they find it very easy to produce a match between the two hemifields by adjusting the intensity of the reference hemifield. On the other hand, such similarity makes it impossible for dichromats to name these stimuli as normal trichromats do. As it will be shown below, this difficulty is greatly reduced when R-G dichromats try to name stimuli with better ecological representativeness than the monochromatic lights used in the Nagel anomaloscope.

Previous research (Lillo et al., 2014) used a set of 102 surface stimuli spanning the full color range to evaluate how accurately R-G dichromats use BCTs and, even more important, to understand what perceptual information they use (Moreira et al., 2014). Participants identified all the stimuli that they could name with a specific BCT. Results indicated an accuracy level far beyond expectations based on the Rayleigh matches performed by the same dichromats. For example, when identifying yellows (see Table III in Lillo et al., 2014) protanopes achieved 75% of hits (pointing to stimuli also pointed by controls as examples of yellow) and only 21% identifying “green” errors (pointing to stimuli that controls pointed as examples of green). Furthermore, looking for yellows did not produce “red” or “orange” errors. Why not if their anomaloscope matches indicate some yellows as identical to some reds and some oranges? The explanation relates to the “psychophysical specificity” concept (Moreira et al., 2014; see also Lillo et al., 2002).

For R-G dichromats, monochromatic stimuli have “low psychophysical specificity”: their perceptual experience in response to a specific combination of wavelength (e.g., 670 nm, the anomaloscope red primary) and intensity (e.g., 20 W) can also be produced by many other wavelength-intensity combinations (e.g., 589 nm and a much lower radiant power for a protanope). On the contrary, some surface stimuli have “high psychophysical specificity” for the same type of dichromat: their perceptual experience with such colors is very difficult or impossible to generate by another surface stimulus. For example, R-G dichromats experience the stimulus usually selected as the best yellow representative (0580-Y in the NCS color atlas, see Lillo et al. (2014), Table A1) with a lightness and saturation (both very high) that no other surface stimulus produces. Table III in Lillo et al. (2014) indicates that protanopes reached 58% of hits when looking for reds (pointing to stimuli also pointed as reds by normal trichromats) and, consequently, only 42% of errors. Most of these errors were “brown” errors (28%, i.e., stimuli pointed were instances of brown, and not of red, for normal trichromats). Something similar happened when protanopes looked for greens. In this case, the percentage of hits was only 46% and, again, most of the errors were “brown” errors (30%). Note that hits and errors derive from the comparison between R-G dichromats and normal trichromats in the pointing task (between-subjects perspective). Very importantly, percentages of “green” and “red” errors were marginal (less than 3%) when both protanopes and deuteranopes looked for, respectively, red and green exemplars (see Tables III and IV in Lillo et al., 2014). This is a very paradoxical result for people that, as previously mentioned, are habitually denominated “R-G dichromats.” This is not to refute that they can confuse some reds with some greens when responding to monochromatic stimuli, similar to the ones used by the anomaloscope, but marginally when responding to representative and ecologically valid surface colors.

With regards to the perceptual information used by R-G dichromats, Moreira et al. (2014) modeled R-G dichromat color naming (hits and errors) using two approaches (models A and B) differing in the number of variables used to define each stimulus and estimate its psychophysical specificity. Model A assumed no activity in the red-green opponent mechanism and, therefore, predicted that stimuli not differing in the activity produced in dichromat’s yellow-blue (variable *s´* in Model A) and achromatic (variable L*_T_ in Model A) mechanisms would be pseudoisochromatic. Most of the R-G dichromats’ naming errors matched the predictions of Model A but, nevertheless, Model A underestimated R-G dichromat naming accuracy because many predicted errors never happened. Model B produced better predictions because it incorporated a new variable (*∆RG_res_*) to estimate some residual activity in the red-green mechanism of R-G dichromats.

Previous works (Lillo et al., 2014; Moreira et al., 2014) have focused on the accuracy of R-G dichromats’ use of BCTs (how well they use each BCT) and distribution of errors (the kind and percentage of errors when looking for exemplars of a given BCT) derived from between-subjects analyses (R-G dichromats vs. normal trichromats). The current study expands this knowledge by including R-G dichromat discriminability (which BCTs are easier or more difficult to differentiate) and metacognition (how valid is the R-G dichromat’s knowledge of their own difficulties) from a within-subjects perspective.

Which is the main difference between BCT accuracy (Lillo et al., 2014; Moreira et al., 2014) and BCT discriminability? Here, accuracy is defined by comparing the performance of dichromats with controls (normal trichromats). This comparison is not required to measure between BCTs’ discriminability. The maximum discriminability level appears when the stimuli identified as members of a BCC (e.g., red) are never identified as members of another one (e.g., yellow or blue) by the observers of the same group (within-subjects perspective). Such result implies that R-G dichromats selectively decide when to use a BCT (and never others) using perceptual information. Of course, this does not imply that the response is accurate, only consistent: e.g., a dichromat can consistently use a BCT (e.g., “red”) to inaccurately name a stimulus that normal trichromats consider an example of another BCC (e.g., brown). Discriminability is reduced when some stimuli are selected as nameable with different BCTs. This result means that there is not enough perceptual information to be sure when a BCT must be used (and not others).

The first goal of the current research relates to discriminability. In order to detail the empirical capacity of R-G dichromats to differentiate pairs of BCTs and to reveal the existence and strength of linkages between BCTs, we reanalyzed confusion matrices from our previous pointing task data (Lillo et al., 2014).

Our second goal relates to dichromat metacognition: how valid is their knowledge about their difficulties. For that purpose, an independent sample of R-G dichromats participated in a verbal task where they evaluated their capacity to discriminate BCTs (metacognition on this capacity, verbal task). The comparison between the results obtained in the pointing task and the verbal task made it possible to know both the similarities and the differences between the practical capacity of R-G dichromats to discriminate between BCTs and their metacognition of their discrimination. Do they know which BCTs they confuse? If so, do they rightly know about the relative magnitude of such confusions? In brief, we are interested in knowing about metacognition in R-G dichromats in relation to their use of BCTs. As it is frequently defined, metacognition (Flavell, 1979) is “cognition about cognition.” That is, knowledge about the cognitive processes and/or their results (Shea et al., 2014). Here, we used a verbal task where R-G dichromats estimated which, and how much, are the BCTs that they think are difficult to differentiate for them, so we are interested in the metacognition in R-G dichromats about the results of perceptive and cognitive processes involved in the use of BCTs. Such estimated difficulties (verbal task) were compared with the empirical confusions measured by their actual discrimination (pointing task, see above). We run MDS analyses to compare the underlying dimensions that allow cognitive discrimination (verbal task) between BCTs with perceptual discrimination (pointing task). We used separate groups of dichromats so that the experience with one task did not influence their performance in the other task.

Our final goal was to analyze the perceptual and cognitive dimensions that supported dichromat performance on the two tasks. This aim was achieved using an individual differences scaling analysis (INDSCAL or dimensional weighting model, see Borg et al., 2013) taking the confusion matrices corresponding to both the pointing and the verbal tasks as input, to examine the relative importance of the dimensions previously revealed by the MDS analyses as a function of the performed task.

## Materials and Methods

### Participants

#### Pointing Task

Seventeen R-G dichromats (8 protanopes, age range 17–36, mean age = 23.5 years; 9 deuteranopes, age range 24–35, mean age = 32.5 years) participated in the pointing task.

#### Verbal Task

Thirty-one R-G dichromats (15 protanopes, age range 20–50, mean age = 34.0 years; 16 deuteranopes, age range 18–51, mean age = 28.06 years) took part in the verbal task.

All the participants of both tasks were males. The color vision of all participants was tested by means of the Ishihara pseudoisochromatic color plates (Ishihara, 2011), the City University Color Vision Test (CUCVT; Fletcher, 1980), the Lanthony test (Lanthony, 1985), and Rayleigh matches on an anomaloscope (Nagel anomaloscope, Tomey AF-1, Tomey, Nagoya, Japan; Birch, 2001). No participant produced tritan responses either in the CUCVT or in the Lanthony tests. All the dichromats accepted the full range of red-green mixtures from the anomaloscope.

All participants were naïve to the experiments’ purposes and had normal or corrected-to-normal visual acuity. The experiment was conducted in accordance with the Declaration of Helsinki and was granted ethical approval by the Hospital Clínico San Carlos Review Board of the Universidad Complutense de Madrid (Spain). All participants collaborated voluntarily in the research and could stop their participation at any time.

### Materials and Stimuli

#### Pointing Task

The stimuli set was composed of 102 stimuli selected from the NCS color atlas (SCI, 1997) on the basis of previous research (Lillo et al., 2007). The stimuli set and the viewing conditions are fully described in Lillo et al. (2014, see Table A1 for the colorimetric specification and the spatial location of the color stimuli included in the set). Stimuli were chosen to include: (i) best exemplars for each BCT, (ii) “boundary-stimuli” between categories and (iii) stimuli halfway along the line in CIELUV space between a best exemplar and each relevant boundary color. Presentation of the 102 stimuli set was arranged simultaneously in a single 15 × 7 matrix (12 columns contained seven samples, and the remaining three columns contained six samples) on a gray background (S 5000-N, L* = 50), with a small gap between adjacent stimuli. Viewed from 50 cm, each stimulus was 4° square, and the entire display was 64.42° × 33.08°. Illuminance was between 225 and 250 lux and correlated color temperature was equal to 5800 K. All measurements were performed using a PR-650 SpectraScan spectrocolorimeter.

#### Verbal Task

A single text table was presented over a white paper with the 11 BCTs in Spanish along both the first column and the first row as headings: *rojo* “red,” *verde* “green,” *azul* “blue,” *amarillo* “yellow,” *rosa* “pink,” *naranja* “orange,” *morado* “purple,” *marrón* “brown,” *blanco* “white,” *gris* “gray,” and *negro* “black” (Lillo et al., 2007, 2018). The vertical axis (first column) indicated the name used by normal trichromats, and the horizontal axis (first row) indicated the name used by the participant. The table contained a matrix of 11 × 11 empty cells (with 121 blank cells in total).

### Procedure

#### Pointing Task

The 102 stimuli were presented simultaneously. The observers were asked to indicate which stimuli were instances of a given BCC. The searching order of the 11 Spanish BCTs varied randomly among observers.

#### Verbal Task

The matrix was presented in front of the experimenter and the observer. The observers were asked to indicate the percentage of confusion between two BCTs (e.g., when red is the name for other observers, which percentage of times you would use the name green?) and the experimenter wrote the values on the matrix. They needed to report a percentage for 110 cells of the matrix (excluding the 11 cells of the main diagonal, when the name for others observers and the name for the tested observers were the same, i.e., the name would have been compared against itself). The matrix rows (name used by normal trichromats) were completed randomly among observers.

## Results

### Confusion Matrices

Tables 1 and 2 summarize the main results provided by the pointing task in relation to BCTs empirical discriminability for protanopes (Table 1) and deuteranopes (Table 2). The diagonals in Tables 1 and 2 show the percentage of specific pointing for each BCT and dichromat type. For example, the diagonal cell located in the upper row of Table 1 indicates that when looking for reds 53.26% of the stimuli pointed to by protanopes was to stimuli never pointed to when looking for examples of any other BCT. Table 1 also informs about non-specific pointing, the pointing done to stimuli also selected when looking for instances of other BCTs. In the case of “red,” the shared pointing was with “green” (11.23%), “black” (4.45%), “brown” (19.56%), and “orange” (8.07%). Tables 1 and 2 do not inform about percentages under 3% (consequently, each row sum can be less than 100%).

**Table 1:** Protanope confusion matrix obtained in the pointing task.

Looked-for BCT	Selected BCT										
	Red	Green	Yellow	Blue	White	Black	Brown	Pink	Orange	Purple	Gray
Red	53.26	11.23				4.45	**19.56**		8.07		
Green	5.11	45.40	3.15				20.16^**^	5.13	**7.66**		**10.23**
Yellow		9.95	61.82		6.56			4.92	**16.74**		
Blue		4.92		48.46				12.82		25.13^***^	5.88
White			6.06		70.71			**19.16**			
Black	6.48			3.33		67.04	13.15			6.67	
Brown	**10.56**	23.92^**^				4.87	51.97		5.59		3.09
Pink		4.59		6.17	**5.89**			53.26		**9.43**	18.32^*^
Orange	6.43	**13.41**	**9.30**				8.25		62.61		
Purple				23.05^***^		3.55		**17.96**		52.78	
Gray		**11.21**		3.47				22.42^*^			58.46

The first column indicates the looked-for BCT. The diagonal shows the percentage of specific pointing (stimuli identified as only belonging to one basic color term (BCT), indicated in the first column). Other values indicate the percentage of non-specific pointing (stimuli identified as belonging to multiple BCTs). Only percentages over 3% are shown. The BCT pairs with a mean link (shared use) higher than 10% (average between both directions of the pair, e.g., Red-Brown 19.56 and Brown-Red 10.56, mean shared use is 15.06%) are in bold. The number of asterisks indicates the strength of the link for the three least discriminable BCT pairs (more asterisks, less discriminability).

**Table 2:** Deuteranope confusion matrix obtained in the pointing task.

Looked-for BCT	Selected BCT										
	Red	Green	Yellow	Blue	White	Black	Brown	Pink	Orange	Purple	Gray
Red	62.60	4.43					12.47	3.13	8.54	7.78	
Green		43.47				3.70	21.03^***^	4.75		4.75	14.14^*^
Yellow		14.49	58.39		3.13		8.81		**12.06**		
Blue		8.59		51.70		4.93	4.74	5.53		**17.58**	6.92
White			3.03		82.65						11.82
Black		16.14		6.32		45.45	**17.61**			8.18	6.29
Brown	4.62	26.35^***^				**5.06**	45.23			4.21	7.11
Pink		6.72						50.21		**11.19**	25.23^**^
Orange	10.52	6.07	**9.94**				6.07		61.28	4.83	
Purple	4.49	9.27		**10.08**		3.66	6.55	**15.41**		37.55	10.11
Gray		16.83^*^					6.75	21.21^**^		6.17	41.30

The first column indicates the looked-for BCT. The diagonal shows the percentage of specific pointing (stimuli identified as only belonging to one BCT, indicated in the first column). Other values indicate the percentage of non-specific pointing (stimuli identified as belonging to multiple BCTs). Only percentages over 3% are shown. The BCT pairs with a mean link (shared use) higher than 10% (average between both directions of the pair, e.g., Green-Brown 21.03 and Brown-Green 26.35, mean shared use is 23.69%) are in bold. The number of asterisks indicates the strength of the link for the three least discriminable BCT pairs (more asterisks, less discriminability).

Tables 3 and 4 summarize the main results provided by the verbal task (metacognition) for protanopes (Table 3) and deuteranopes (Table 4). Diagonals are empty because observers did not estimate BCT specificity (percentage of correct use of the category in relation to normal trichromat use), but only which BCTs (and how much) they confused. As it will be shown, we used the results of Tables 1–4 to measure between BCT discriminability in two different ways: “less discriminable pairs” (Table 5) and “best discriminable pairs” for each BCT (empty cells in Tables 1–4).

**Table 3:** Protanope confusion matrix obtained in the verbal task.

Target BCT	BCT used by protanopes										
	Red	Green	Yellow	Blue	White	Black	Brown	Pink	Orange	Purple	Gray
Red		4.34					**19.50**	10.33	6.53		
Green	7.40		26.67^*^	6.73			41.17^***^		**20.13**		5.40
Yellow		26.00^*^							9.33		
Blue		7.13						11.73		35.00^**^	5.20
White											
Black											
Brown	**22.67**	39.33^***^							3.47		
Pink	7.80			7.60						**13.20**	**13.20**
Orange	9.67	**16.33**	4.67				3.43				
Purple	4.67			41.00^**^				**23.33**			
Gray		8.73		4.47				**15.67**			

The first column indicates the target BCT (name used by normal trichromats). The diagonal is empty because participants did not estimate the percentage of target BCTs’ specificity, but only the percentage of confusion between BCTs. Only percentages over 3% are shown. The BCT pairs with a mean link (percentage of confusion) higher than 10% (average between both directions of the pair, e.g., Red-Brown 19.50 and Brown-Red 22.67, mean percentage of confusion is 21.09%) are in bold. The number of asterisks indicates the strength of the link for the three least discriminable BCT pairs (more asterisks, less discriminability).

**Table 4:** Deuteranope confusion matrix obtained in the verbal task.

Target BCT	BCT used by deuteranopes										
	Red	Green	Yellow	Blue	White	Black	Brown	Pink	Orange	Purple	Gray
Red		4.56					19.81^*^	**8.75**	**18.19**	6.13	
Green	6.44		8.75				37.31^***^				7.13
Yellow		7.69							**13.56**		
Blue						8.25				23.50^**^	
White											
Black				5.81							
Brown	20.94^*^	33.94^***^	4.75						5.56		
Pink	**16.13**				4.50					7.00	6.88
Orange	**18.63**		**10.13**				4.63				
Purple	5.38			33.44^**^				8.56			
Gray		4.19						6.31			

The first column indicates the target BCT (name used by normal trichromats). The diagonal is empty because participants did not estimate the percentage of target BCTs’ specificity, but only the percentage of confusion between BCTs. Only percentages over 3% are shown. The BCT pairs with a mean link (percentage of confusion) higher than 10% (average between both directions of the pair, e.g., Red-Brown 19.81 and Brown-Red 20.94, mean percentage of confusion is 20.38%) are in bold. The number of asterisks indicates the strength of the link for the three least discriminable BCT pairs (more asterisks, less discriminability).

**Table 5:** Less discriminable pairs of BCTs.

Pair	Protanope pointing	Deuteranope pointing	Protanope verbal	Deuteranope verbal
Red-Brown	15.06 (4)		21.09 (4)	20.38 (3)
Red-Orange				18.41 (4)
Red-Pink				12.44 (5)
**Green-Brown**	**22.04 (2)**	**23.69 (1)**	**40.25 (1)**	**35.63 (1)**
Green-Gray	10.72 (8)	15.49 (3)		
Green-Orange	10.53 (9)		18.23 (6)	
Green-Yellow			26.34 (3)	
Yellow-Orange	13.02 (6)	11.00 (7)		11.65 (6)
**Blue-Purple**	**24.09 (1)**	**13.83 (4)**	**38. 00 (2)**	**28.47 (2)**
White-Pink	12.53 (7)			
Black-Brown		11.34 (6)		
Pink-Purple	13.70 (5)	13.30 (5)	18.27 (5)	
Pink-Gray	20.37 (3)	23.22 (2)	14.44 (7)	

Pairs of BCTs with mean shared use (pointing task) or mean percentage of confusion (verbal task) over 10% (shown in bold in Tables 1–4) for at least one of the four dichromat type-task possible combinations: Protanope Pointing, Deuteranope Pointing, Protanope Verbal, and Deuteranope Verbal. Note that only 13 BCT pairs out of the total of 55 possible pairs fulfilled the 10% criterion of high confusion in at least one dichromat type-task combination. The two BCT pairs that fulfilled the 10% criterion for the four dichromat-task possible combinations are in bold. The values in brackets indicate the order of confusion of those pairs within each dichromat type-task combination (e.g., Blue-Purple is the less discriminable pair in the protanope pointing task, therefore its percentage appears next to number 1 in brackets; Green-Brown is the less discriminable pair in the deuteranope pointing task, therefore its percentage appears next to number 1 in brackets).

Tables 1–4 percentages in boldface correspond to the “less discriminable BCT pairs” (the number of asterisks indicates the strength of the link of the three less discriminable BCT pairs: more asterisks, less discriminability). That is, pairs with mean shared use over 10%. For example, Table 1 shows that the 10% criterion was fulfilled by the Green-Orange pair in protanopes because 7.66% of the stimuli pointed to when looking for greens were stimuli also pointed to when looking for oranges and, complementarily, 13.41% of the stimuli pointed to when looking for oranges were stimuli also pointed to when looking for greens. The mean value between 7.66 and 13.41 is 10.53. This mean value for the Green-Orange pair appears in the Table 5 together with the other pairs fulfilling the 10% criterion.

Table 5 highlights that there were only 13 BCT pairs out of the total of 55 possible pairs that fulfilled the 10% criterion for at least one group of dichromats (protanopes or deuteranopes) in one task (pointing or verbal). Only two of these pairs, Green-Brown and Blue-Purple, were over the 10% criterion for every type of dichromat-task combination. The comparison of pairs of columns of Table 5 allows the specification of two kinds of concordances. The first one when the 10% criterion is fulfilled in both columns. This is what happens, for example, in the case of Green-Brown in the pointing task for protanopes and deuteranopes, as shown by the percentages of 22.04 and 23.69%. The second one when the 10% criterion is not fulfilled in any of the columns, so they appear as empty cells. This is what happens, for example, in the case of Red-Orange in the pointing task for protanopes and deuteranopes. In contrast, a discrepancy exists when the 10% criterion is only fulfilled in one column, as in the case of Red-Brown in protanopes (15.06%) and deuteranopes (empty cell) in the pointing task. Table 5 indicates that there were only four discrepancies between protanopes and deuteranopes in the pointing task and seven in the verbal task. Table 5 also indicates that there were only four discrepancies between the pointing and the verbal task in protanopes and seven in deuteranopes. Of course, it must be kept in mind that Table 5 only represents the 13 BCT pairs out of the total of 55 possible pairs that fulfilled the 10% criterion in at least one type of dichromat-task combination, therefore, the 42 remaining pairs not indicated in Table 5 are also coincidences between protanopes and deuteranopes (i.e., pairs under the 10% criterion both for protanopes and deuteranopes for both tasks, which would be recorded as four white cells if represented in Table 5).

Table 5 indicates, by brackets, the order of confusion within each dichromat type-task combination (e.g., Blue-Purple is the less discriminable pair in the protanope pointing task, therefore, its percentage appears next to number 1 in brackets; Green-Brown is the less discriminable pair in the deuteranope pointing task, therefore its percentage appears next to number 1 in brackets).

Finally, to end with the descriptive analysis of the confusion matrices, we will focus on the number of empty cells included in each row of Tables 1–4, which correspond to the best discriminable pairs for each dichromat type-task combination. For example, Table 1 shows that when protanopes looked for whites in the pointing task only two other BCTs produced shared use: “yellow” (6.06%) and “pink” (19.16%). So, the number of best discriminable BCTs related to white was eight (10 categories different to white minus 2 is equal to 8). The result was different for protanopes in the verbal task (Table 3): They estimated that there were no other BCT that could be confused with white and, consequently, now the number of empty cells reaches its maximum (10 minus zero is equal to 10). Wilcoxon non-parametric tests were performed to compare the number of empty cells corresponding to the different BCTs between different combinations of type of dichromat‐ type of task. These analyses indicated that the number of empty cells in the pointing task was significantly lower than in the verbal task both for protanopes and for deuteranopes (*Z* = −2.06, *p* < 0.05 and Z = −2.63, *p* < 0.01, respectively). Wilcoxon tests also indicated that there were no significant differences between protanopes and deuteranopes in the number of empty cells in neither the pointing task (*Z* = −1.22, *p* = 0.222) nor in the verbal task (*Z* = −0.88, *p* = 0.380).

### MDS Analyses

The complete confusion matrices derived from the pointing and the verbal tasks (Tables 1–4 omit percentages under 3%) were used as input for multidimensional scaling (MDS) analyses to reveal the dimensions underlying color naming and its metacognition in R-G dichromats. Contrary to the representation of normal trichromat color naming, whose MDS solutions show three relevant dimensions (that resemble the activity of the red-green, yellow-blue and achromatic mechanisms), the 11 BCTs in R-G dichromats are properly represented on color planes corresponding to 2D MDS solutions that clearly show one chromatic dimension (with blue and orange at the ends) and an achromatic dimension (with white and black at the ends; Moreira, 2010; Lillo et al., 2014).

In order to compare the MDS solutions derived from the verbal and the pointing tasks, we performed the same MDS analyses as used by Lillo et al. (2014). Therefore, we conducted non-metric MDS analyses using Proxscal in SPSS entering exactly the same parameters in the analyses as in the previous publication (Proxscal minimizes the normalized raw stress value, which is a measure of departure from goodness of fit ranging from 0 to 1: the smaller this value, the better the fit). The input for these MDS analyses were the confusion matrices obtained in the verbal task for protanopes (Table 3) and deuteranopes (Table 4; complete matrices were used, as stated before). The fit for the bidimensional solutions was good (normalized raw stress 0.0312 for protanopes and 0.0213 for deuteranopes), and remarkably comparable to the fit for the bidimensional solutions obtained using the confusion matrices derived from the previous MDS analyses for the pointing task (normalized raw stress 0.0233 for protanopes and 0.0282 for deuteranopes, see Lillo et al., 2014).

**Figure 1:**
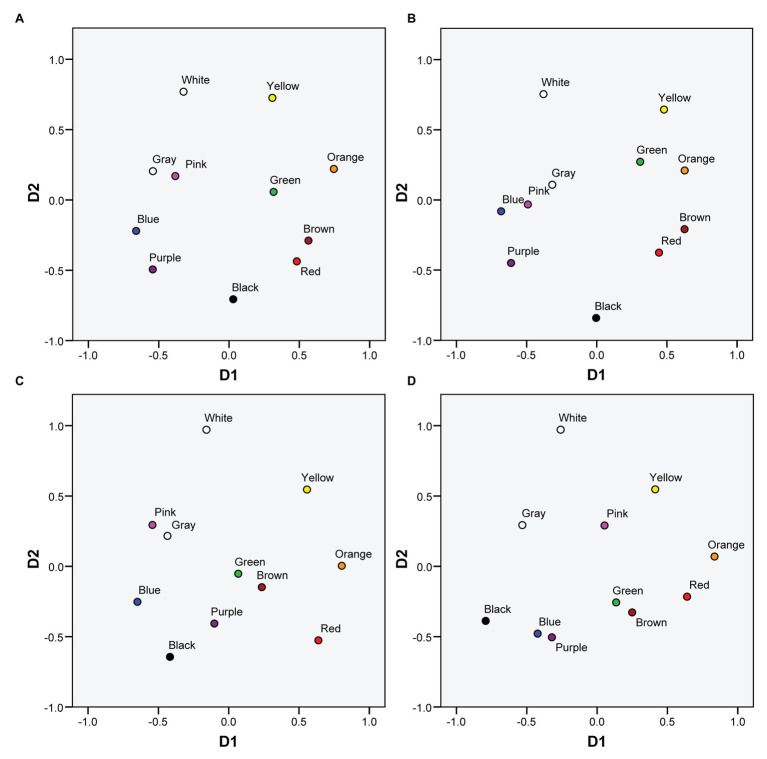
Color planes corresponding to multidimensional scaling (MDS) solutions for protanopes and deuteranopes derived from the mapping and the verbal tasks. **(A)** Protanopes, pointing task. **(B)** Protanopes, verbal task. **(C)** Deuteranopes, pointing task. **(D)** Deuteranopes, verbal task. **(A,C)** Colored versions of Figures 3A,B in Lillo et al. (2014; reproduced with permission). **(B,D)** Represent the transformations applied to the original MDS solutions obtained from the verbal task in order to maximize the global correlation between the 2D MDS solutions derived from both tasks (see text for details).

**Figure 2:**
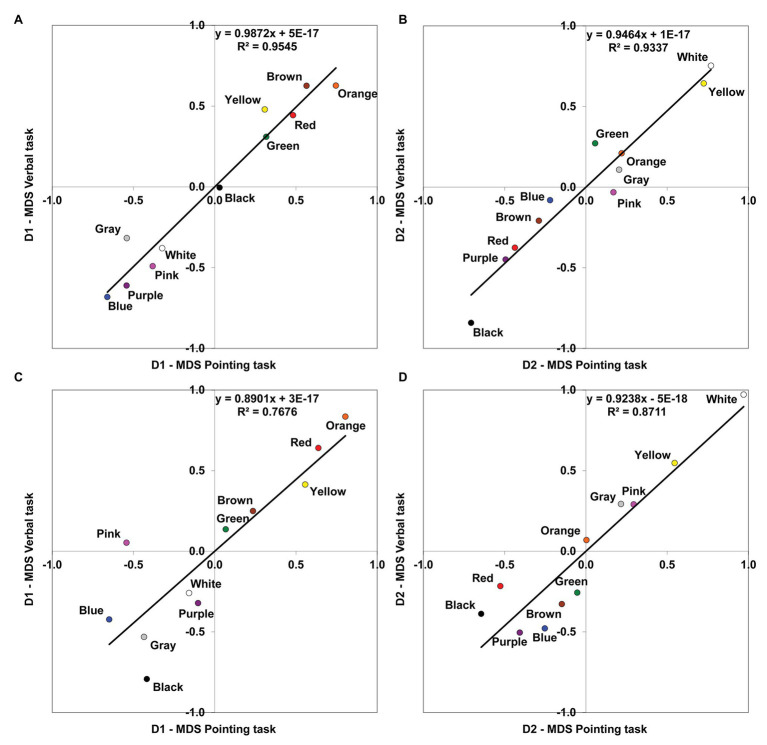
Comparison of MDS solutions derived from the pointing and the verbal tasks. Linear relationships are shown between the corresponding dimensions (D1, chromatic; D2, achromatic) obtained in the MDS analyses for the two different tasks. **(A)** Protanopes, D1. **(B)** Protanopes, D2. **(C)** Deuteranopes, D1. **(D)** Deuteranopes, D2. The continuous line represents the least squares linear regression fit (the corresponding equations and *R*^2^ values are shown in each graph).

Figure 1 represents the MDS solutions both for protanopes and deuteranopes derived from the pointing and the verbal tasks. Figure 1A (protanopes, pointing task) and Figure 1C (deuteranopes, pointing task) are colored versions of Figures 3A,B in Lillo et al. (2014). We reproduce these figures here to facilitate the visual comparison of MDS solutions derived from the pointing and the verbal tasks. Figures 1B,D (protanopes, deuteranopes) represent the MDS solutions derived from the verbal task. The MDS solutions obtained directly from the verbal task were reflected and rotated (these transformations preserve the geometric shape of the configuration, see, for example, Borg et al., 2013) in order to maximize the global correlation between the 2D MDS solutions derived from both tasks (see Figure 2). Specifically, dimension 1 (D1, with orange and blue located near the ends) was reflected both for protanopes and deuteranopes, and the global solution was slightly rotated 25.4° counterclockwise for protanopes and 30.3° counterclockwise for deuteranopes.

As it can be seen, apart from the global similarity between the four color planes represented in Figure 1 (the locations and distances between BCTs tend to be very similar), there is a high concordance between the color planes derived from both tasks, for both protanopes (Figures 1A,B) and deuteranopes (Figures 1C,D). This global similarity is illustrated and quantified in Figure 2, which represents the comparison of the MDS solutions derived from the pointing and the verbal tasks.

Figure 2 represents the MDS solution derived from the verbal task as a function of the MDS solution derived from the pointing task, with the chromatic dimension (D1) and achromatic dimension (D2) compared separately. The left panel of Figure 2 shows the linear relationship between the first chromatic dimension (D1) obtained in the pointing and the verbal tasks both for protanopes (Figure 2A) and deuteranopes (Figure 2C), and the right panel shows the linear relationship between the second achromatic dimension (D2) obtained in the pointing and the verbal tasks both for protanopes (Figure 2B) and deuteranopes (Figure 2D). For each graph, the equation derived from the linear regression analysis along with the proportion of variance (*R*^2^) is indicated. *R*^2^ values can be interpreted as the proportion of variance in the location of BCTs along the dimensions obtained in the MDS solution as derived from the verbal task which is accounted for by the location of the same BCTs along the corresponding dimension obtained in the MDS solution derived from the pointing task. All the obtained *R*^2^ values ranged from 0.768 to 0.954 and were highly significant (*R*^2^ = 0.954, *F*(1,9) = 188,69, for D1 and *R*^2^ = 0.934 *F*(1,9) = 126,81, for D2 in protanopes; *R*^2^ = 0.768 *F*(1,9) = 29,72, for D1 and *R*^2^ = 0.871 *F*(1,9) = 60,84 for D2 in deuteranopes; all *p* < 0.001).

### INDSCAL Analyses

Figures 1, 2 show that the 2D MDS solutions derived from the pointing and the verbal tasks are qualitatively and quantitatively very similar, but they do not demonstrate the relative importance of the chromatic (D1) and the achromatic (D2) dimensions when protanopes and deuteranopes perform these tasks (pointing and verbal). In order to compare the relative salience of the dimensions underlying color naming and its metacognition in R-G dichromats we performed an INDSCAL (dimensional weighting model, Borg et al., 2013) using the complete confusion matrices corresponding to both tasks and to both dichromat groups as input.

Individual differences scaling gives a common space or global solution with different weights to the dimensions obtained in that common space for each one of the cases (matrices) introduced in the analysis. The weights of the dimensions are usually represented as vectors or points (for the case of 2D MDS solutions, in the plane formed by the weights of the two dimensions). The module of the vectors (the distance from the origin to the end of the vector) indicates the fit of the individual data to the global solution, and the phase angle of the vector (angle formed between the vector and the x-axis) is an excellent way to quantify the relative importance of the dimensions for different individuals with the advantage that it reduces the bidimensionality of the weights to only one dimension. This makes the solution very easy to interpret: Lower phase angles indicate over-weights of D1 relative to D2, whereas higher phase angles indicate over-weights of D2 relative to D1. It is very important to keep this in mind, since the direct comparison of the weights, even for the same dimension, can be totally misleading (consider, for example, the case of two vectors with different modules but the same phase angle: the higher the module, the higher the weights of both D1 and D2, but the relative importance of the dimensions is exactly the same in both cases, as indicated by the phase angle).

**Figure 3:**
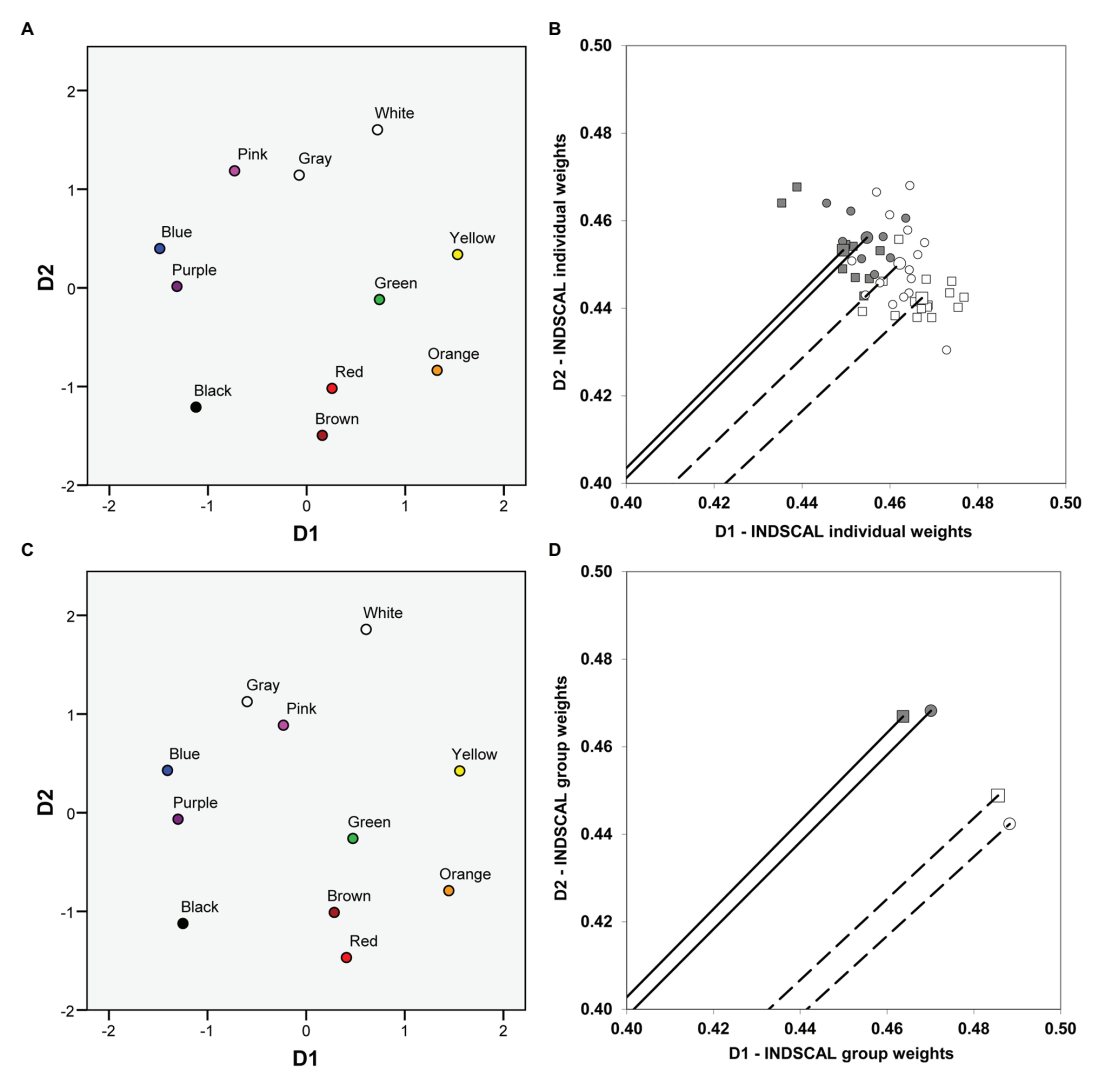
Color planes and dimensional weights obtained in the individual differences scaling (INDSCAL) analyses. The color planes corresponding to the common spaces obtained in the INDSCAL analyses are shown. **(A)** Individual data. **(C)** Group data. The weights for D1 (chromatic) and D2 (achromatic) obtained in the INDSCAL analyses for protanopes (circles) and deuteranopes (squares) in the pointing task (dark gray) and the verbal task (white) are shown. **(B)** Individual data. **(D)** Group data. Note that axes have been truncated for clarity purposes. Solid lines (pointing task) and dashed lines (verbal task) represent the mean vector (centroid) of individual weights **(B)** and the corresponding vectors of group weights **(D)**.

We first used the four group confusion matrices (two groups of dichromats performing the two different tasks: Tables 1–4, complete matrices were used, as stated before) and then repeated the analysis using the 48 individual confusion matrices (17 matrices derived from the pointing task and 31 matrices derived from the verbal task). We obtained exactly the same pattern of results both for group and individual data [the fit, as indicated by the normalized raw stress value, was better for group (0.0405) than for individual (0.0898) data]. The common space (i.e., the global solution for protanopes and deuteranopes in the verbal and the pointing task), represented on the left panel of Figure 3 (Figure 3A: individual data, Figure 3C: group data), again clearly revealed one chromatic dimension (D1, with blue-purple and orange-yellow at the ends) and another achromatic dimension (D2, with white and black-brown at the ends), very similar to the color planes represented in Figure 1. The weights of the dimensions, represented on the right panel of Figure 3, allowed us to compare the relative salience of D1 and D2.

Although the weights between dimensions cannot be fairly compared intra-individually, the order of the dimension weights that different persons have for different dimensions can be compared (Borg et al., 2013). There was a clear tendency toward an equally distributed order of weights in the pointing task (D1 was given more weight than D2 for five out of eight protanopes and for five out of nine deuteranopes) and a clearly biased distribution of the order of weights in the verbal task (D1 was given more weight than D2 for 12 out of 15 protanopes and for all of the 16 deuteranopes).

Figures 3B,D represent the weights of D1 and D2 obtained in the INDSCAL analyses performed on individual (Figure 3B) and group (Figure 3D) data both for protanopes (circles) and deuteranopes (squares) in the pointing (dark gray symbols) and the verbal (white symbols) tasks. Figures 3B,D show that the relative weight of D1 is slightly higher in the verbal task (white symbols) than in the pointing task (dark gray symbols) both for protanopes (circles) and deuteranopes (squares), and the reverse is true for D2, as can be seen by the lower phase angle of the vectors representing the dimensional weights in the verbal task and also by the values of the coordinates. The mean phase angle of the group vectors (Figure 3D) corresponding to the white (verbal task) and the dark gray (pointing task) symbols was 42.46° and 45.04°, respectively; the direct comparison of the coordinates of the weights (D1 or D2) is justified here because the modules of the vectors were nearly equal for group data, 0.66 (otherwise this comparison could be misleading, since the higher the module, the higher the weights of both D1 and D2, as stated before).

Figure 3B shows the individual dimensional weightings. For clarity, only the mean vector (centroid) of the INDSCAL weights obtained for each one of the four type of dichromat-task combination is represented, hence only four vectors instead of 48 are shown. Individual weights are represented as points. As in the case of group data (Figure 3D), the pattern of individual dimensional weightings shows that the relative weight of D1 is slightly higher (the phase angle is lower) in the verbal task (white symbols) than in the pointing task (dark gray symbols) both for protanopes (circles) and deuteranopes (squares), and the reverse is true for D2.

As it has been stated before, the modules of the vectors indicate the fit of the individual data to the common space. A two-way ANOVA with group (protanopes and deuteranopes) and task (pointing and verbal) was conducted on modules to test any possible effect on the fit of the individual spaces to the common space (normality of modules was confirmed by Kolmogorov-Smirnov tests for each type of dichromat-task combination, all *p* > 0.05). This analysis (identical results are found if normalized raw stress values are used instead of modules) confirmed a significant main effect of group [*F*(1,44) = 4.97, *p* < 0.05, *η*^2^ = 0.10], slightly larger modules, hence slightly better individual fit to the global solution, in protanopes (mean module 0.645, normalized raw stress value 0.085) than in deuteranopes (mean module 0.641, normalized raw stress value 0.096). No significant main effect of task or interaction between group and task was found (*p* > 0.05).

**Figure 4:**
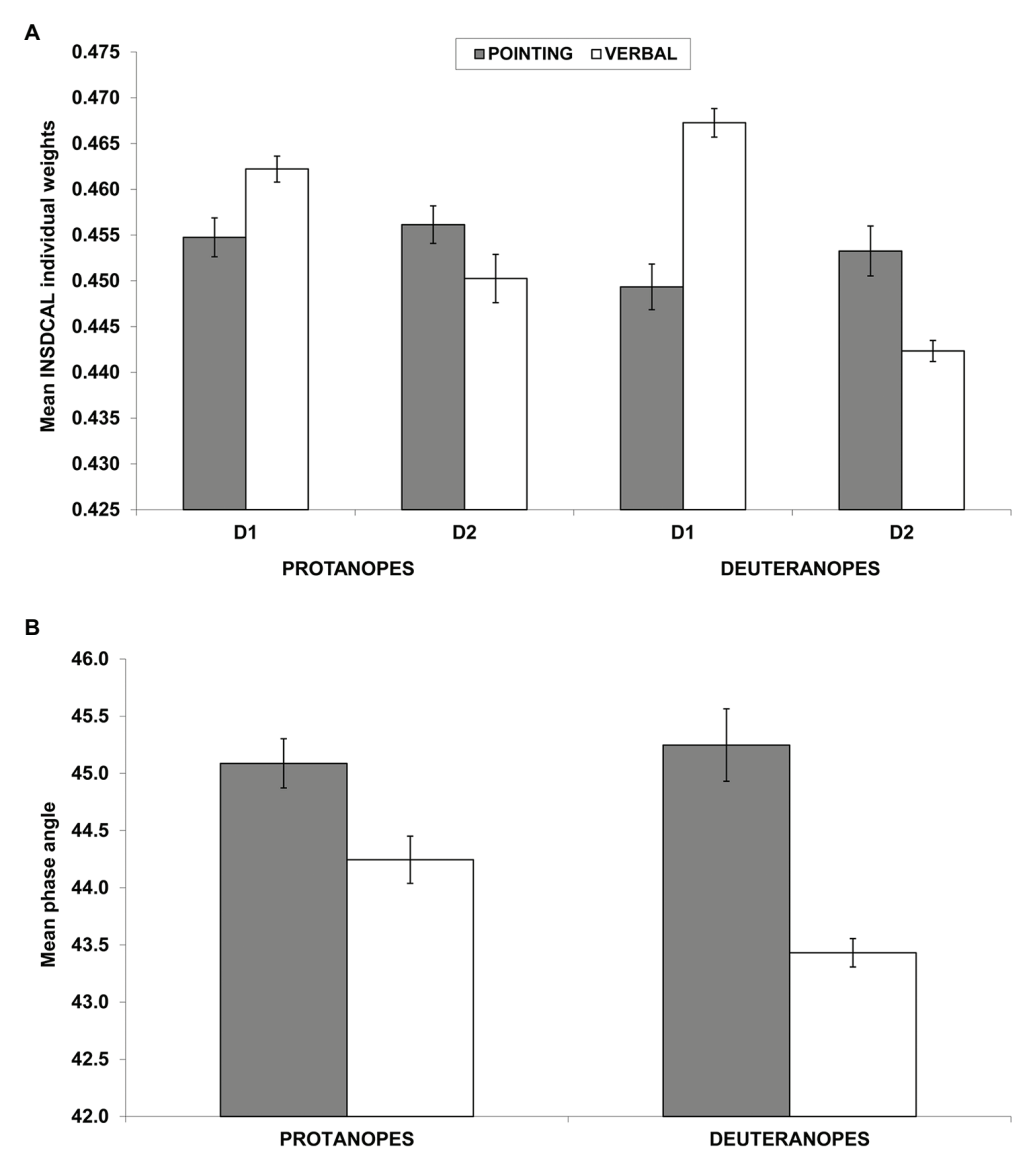
Individual dimensional weights obtained in the INDSCAL analysis **(A)** and phase angles of the corresponding vectors **(B)**. **(A)** Mean (±SEM) weights obtained in the INDSCAL analysis for protanopes and deuteranopes in the pointing task (dark gray bars) and the verbal task (white bars) for D1 (chromatic dimension) and D2 (achromatic dimension). **(B)** Mean (±SEM) phase angles of the weighting vectors obtained in the INDSCAL analysis for protanopes and deuteranopes. Lower phase angles indicate a relative over-weighting of D1 (chromatic dimension) in relation to D2 (achromatic dimension). Note that *y*-axis has been truncated for clarity purposes in both graphs.

Figure 4A shows the mean weights of D1 and D2 for protanopes and deuteranopes in the pointing (dark bars) and the verbal (white bars) tasks. The pattern of results clearly shows the salience of the chromatic dimension (D1) in the verbal task (white bars) compared to the pointing task (dark gray bars), and the opposite trend for the achromatic dimension (D2). The same pattern can be seen for both protanopes and even more clearly for deuteranopes. However, as discussed before, it is possible that some differences in the modules of the individual vectors of protanopes and deuteranopes (see Figure 3D and the results of the ANOVA described above) calls the validity of this interpretation into question. To address this possible problem, Figure 4B shows the mean phase angles both for protanopes and deuteranopes in the pointing (dark bars) and the verbal (white bars) tasks. The pattern of results clearly confirms the interpretation given above, i.e., the salience of the chromatic dimension (D1, lower phase angle) in the verbal task (white bars) compared to the pointing task (dark gray bars) both for protanopes and deuteranopes.

A two-way ANOVA with group (protanopes and deuteranopes) and task (pointing and verbal) was conducted on phase angles (normality of phase angle was confirmed by Kolmogorov-Smirnov tests for each type of dichromat-task combination, all *p* > 0.05). This analysis confirmed a significant main effect of task [*F*(1,44) = 37.94, *p* < 0.001, *η*^2^ = 0.463], with lower phase angles (therefore, more salience of chromatic dimension, D1) for the verbal task (43.84°) in relation to the pointing task (45.17°) and a significant interaction between group and task [*F*(1,44) = 5.09, *p* < 0.05, *η*^2^ = 0.10]. Tukey *post-hoc* comparison on the group*task interaction indicated that this task effect is slightly stronger in deuteranopes than in protanopes, as it showed lower phase angles for deuteranope verbal (43.43°) in relation to protanope verbal [44.24°, *p* < 0.05; the task main effect was also confirmed both for protanopes (44.24° vs. 45.09°, *p* < 0.05) and deuteranopes (43.43° vs. 45.25°, *p* < 0.01)]. No significant main effect of group was found [*F*(1,44) = 2.29, *p* = 0.138; 44.66° for protanopes, 44.34° for deuteranopes], contrary to what was found in the previous ANOVA conducted on modules.

That is, it seems that the metacognition of the use of BCTs in R-G dichromats (as revealed by the verbal task), is slightly more driven by a chromatic dimension and slightly less driven by an achromatic dimension than their practical use of BCTs (as revealed by the pointing task), specially for deuteranopes.

## Discussion

Red-Green dichromats’ metacognition about their difficulties using BCTs is not perfect, and can be considered a caricature of their practical difficulties. As in the case of a caricature of a face: (1) This knowledge includes fewer confusions between BCT pairs (just as caricatures will include less detail in facial features), and (2) the magnitude of the most important difficulties are exaggerated (just as the most salient facial features in a caricature are oversized). These two ideas are supported by the following facts. Firstly protanopes and deuteranopes reported more BCT pairs as easy to differentiate (represented by more empty cells in the verbal task, Tables 3 and 4, than in the pointing task, Tables 1 and 2), while, at the same time, and secondly R-G dichromats’ metacognition overestimated the magnitude of the difficulties for the two more problematic pairs. Specifically, as Table 5 indicates, empirical confusions between Green-Brown occurred 22.04% of the times for protanopes and 23.69% for deuteranopes, and empirical confusions between Blue-Purple occurred 24.09% of the times for protanopes and 13.83% for deuteranopes. On the other hand, Table 5 also indicates that the corresponding confusions estimated for these pairs were 40.25% (Green-Brown) and 38.00% (Blue-Purple) for protanopes, and 35.63% (Green-Brown) and 28.47% (Blue-Purple) for deuteranopes.

Despite its overstated nature, protanopes’ and deuteranopes’ metacognition about their capacity to differentiate BCTs is very accurate, as showed by the strong concordance between the results derived from the pointing and the verbal tasks. It must be emphasized that these tasks were performed by different groups of observers, so our experimental design avoided the possibility of mutual influence. Consequently, it can be concluded with confidence that people referred to as “R-G” dichromats (see, for example, Kaiser and Boynton, 1996, chapter 10; Baraas et al., 2010) have a very accurate knowledge on which BCT pairs they have most difficulty differentiating, which paradoxically, do not include the Red-Green pair.

The accuracy of protanope and deuteranope metacognition was found by comparing their empirical and estimated confusions between BCTs (Table 5, see also Tables 1–4), the number and identity of the most discriminable BCTs pairs (empty cells in Tables 1–4), and the similarity of the location of the BCTs on the color planes derived from MDS analyses (Figures 1, 2). With INDSCAL analysis (Figures 3, 4) however, there was a change in the relative relevance of the chromatic (D1) and the achromatic (D2) dimensions depending on the task, especially in the case of deuteranopes: D1 was more relevant for metacognition (smaller mean phase angle values) than for BCTs’ empirical use.

A closer look at which difficulties in differentiating BCTs posed the greatest problems for the R-G dichromats tested (see Table 5), indicates that there were 13 less discriminable pairs (those over the 10% criterion in either the pointing or the verbal task for, at least, one group of dichromats). This number is way below 55, the theoretically possible combinations of pairs, and reduces to nine (protanopes) or seven (deuteranopes) when considering only the BCT pairs over the 10% criterion in the pointing task. Six of the less-discriminable pairs in this task are the same both for protanopes and deuteranopes (Green-Brown, Green-Gray, Yellow-Orange, Blue-Purple, Pink-Purple, and Pink-Gray) with some pairs specific to each dichromat type (Red-Brown, Green-Orange, and White-Pink for protanopes, and Black-Brown for deuteranopes). This is a very important result, because it clearly indicates that the so called “R-G” dichromats have a relatively good capacity to differentiate BCTs (Tables 1 and 2), only demonstrating difficulties in differentiating nine (protanopes) or seven (deuteranopes) out of a total of 55 possible pairs. This finding complements our previous work on the accuracy of dichromat BCT use (Lillo et al., 2014; Moreira et al., 2014), where we found that dichromats frequently categorize stimuli correctly, using the same BCTs as normal trichromats (making fewer errors than predicted by the standard model of dichromacy).

When comparing the empirical difficulties in differentiating BCTs (pointing task) with the dichromat’s metacognition (verbal task), there are two very special pairs: Green-Brown and Blue-Purple (rows in boldface in Table 5). Only these pairs were found to be very difficult for both dichromat types to differentiate both empirically and in metacognition. Considering this, and taking into account the dichromats’ minor difficulties in differentiating the Red-Green pair (Tables 1–4), why not refer to them as “Blue-Purple” or, even better, “Brown-Green” dichromats? As it will be shown, this last choice has two important advantages.

As indicated by the three asterisks in Tables 3 and 4, the Green-Brown pair was evaluated as the most difficult one by both protanopes and deuteranopes. This fact mirrors the ease with which examples of Green-Brown confusion can be found in everyday situations (see, for example, Fletcher and Voke, 1985, chapter 11; Birch, 2001, chapter 11; McIntyre, 2002) and facilitates the dichromats’ self-identification as Green-Brown dichromats, which cannot be said for the Red-Green pair. Second, an even more important advantage, “brown” is a term that is suitable for “related colors” (Shevell, 2003, chapter 4; Hunt and Pointer, 2011, chapter 1 and appendix 9) but not for “unrelated colors.” This specificity does not apply to most BCTs (i.e., “red,” “green,” “blue,” etc.) which can be used for naming unrelated colors (see Paramei et al., 1998).

The colors experienced when looking at points of light are examples of unrelated colors. They can have a given brightness level, but they lack a lightness value (the terms “light” or “dark” are not applicable). The stimuli used in some very influential color vision tests (e.g., the Nagel anomaloscope) are unrelated colors that make it easy to find instances of reds and greens that are not differentiable by protanopes and deuteranopes. So, to name these people, “Red-Green” dichromats (hereinafter referred to as Brown-Green dichromats) are fully accurate only when considering unrelated colors. But this type of stimuli is very infrequent in everyday environments and, consequently, is unlikely to influence how BCTs are acquired (Franklin, 2015). On the contrary, such acquisition must be based on everyday interactions with related colors because of their preeminence in everyday contexts. Related colors became the primary stimuli type in the study of BCTs for this same reason (see Berlin and Kay, 1969; Kay et al., 2009; Lillo et al., 2018).

Our results show an important degree of agreement between protanopes and deuteranopes both for the descriptive analysis performed on the confusion matrices (Tables 1–5) and the pattern of results derived from the MDS analyses: key similarities can be seen between protanopes and deuteranopes’ color spaces (Figure 1) and the two dimensions (Figure 2) defining such spaces. As a corollary to our commentary on the many concordances found between protanopes and deuteranopes, we take the gamble that they may be related to the most famous case of color vision diagnostic error, i.e., the case of John Dalton himself. For two centuries he was considered a protanope due to his metacognition (i.e., his descriptions of his experience) about the color appearance of objects and their similarities and dissimilarities (Dalton, 1798; see also Fletcher and Voke, 1985, chapter 5). The genetic analysis performed at the end of the 20th century (Hunt et al., 1995) indicated that he was really a deuteranope. It is likely that diagnostic error was facilitated by the similarities between both types of Brown-Green dichromats’ metacognition (see Figures 1B,D).

One of the most surprising facts discovered was that the change in the relative weights of the two dimensions derived from the INDSCAL analysis was task dependent. As it is indicated by the differences between the dark gray (pointing task) and the white bars (verbal tasks) in Figure 4B, the chromatic dimension (D1 in Figure 3) was more dominant in the explanation of BCT differences than the achromatic dimension (D2) in the verbal task (lower phase angle values in Figures 3B, 4B). This difference was especially marked with the deuteranope group in which every participant (16 out of 16) gave higher weight to D1 than to D2.

Two instances regarding the use of “black” and “white,” the two ends of the achromatic dimension, concord with this change in the relative importance of D2. Firstly, the reduced values (near to zero) produced in the verbal task when dichromats estimate their difficulty differentiating either “black” or “white” from any other BCT. For the verbal task, “white” has 10 empty cells recorded both for protanopes and deuteranopes. This is the maximum possible and indicates that, according to dichromat’s metacognition, they have no difficulty differentiating “white” from any other BCT. A similar result was reported for “black,” for which the number of empty cells for protanopes and deuteranopes were, respectively, 10 and 9. No other BCT produced so many empty cells in the verbal task. Second, we will explain how a possible bias may have been introduced by the criteria we used to create our pointing task stimuli set.

Because of the great number of discriminable colors (more than 2 million, Linhares et al., 2008a; Kuehni, 2016), some more or less arbitrary criteria must be used to decide which stimuli to include in any color set. For example, the 330 stimuli used in the Word Color Survey (WCS set, Berlin and Kay, 1969; Kay et al., 1997, 2009) were chosen by selecting Munsell colors: (1) with the maximum saturation for each available hue-lightness combination, and (2) included a gray scale (from white to black). For previous research (Lillo et al., 2014), we decided not to use this set because it includes too few low saturation colors, which were of the most interest as these stimuli produce more categorization problems for Brown-Green dichromats. Instead, we used the results from a previous work (Lillo et al., 2007) to create a 102 stimuli sample including: (1) the best exemplar for each BCC, (2) stimuli at the boundary between two BCC, and (3) stimuli halfway between each best exemplar and category boundary stimuli. As expected, this set produced a significant number of dichromat naming errors, making it possible to develop a very accurate explicative model (Moreira et al., 2014, model B) of the psychophysical information used by dichromats to name color stimuli. One criticism is that our set may relatively overrepresent those BCCs that, like white and black, occupy small volumes in CIE color space, and underrepresent the BCCs that occupy large volumes (like green and brown). A way to compensate for this possible bias is to adjust the number of halfway stimuli (3) according to each BCT volume.

It is very interesting to analyze our results in the framework provided by the debate between the universalistic (UE, Berlin and Kay, 1969; Kay and Maffi, 1999; Kay et al., 2009) and the relativistic (LRH, Saunders and van Brakel, 1997; Davidoff et al., 1999; Roberson et al., 2000; Davidoff, 2015) theories. This analysis must take into consideration the difference between our experimental design and those most commonly used in this area of research: instead of comparing people with similar perceptual characteristics but differing in language (e.g., Lin et al., 2001; Goldstein et al., 2009; Brown et al., 2016), we studied BCT use in people socially pressed to use the same set of BCTs as normal trichromats despite their reduced color gamut. Against what was expected, the dichromats did achieve many accurate BCT discriminations (42 BCT pairs are missing in Table 5), using this capacity to accurately name most color stimuli (Lillo et al., 2014) and, the last but not least, they reached a good level of knowledge (metacognition) about their capacities and limitations. A superficial analysis of these results could wrongly lead one to conclude that they are only compatible with the LRH (Roberson et al., 2000; Davidoff, 2015), in the sense that the internal color space is a “tabula rasa” where the linguistic-cultural factors freely segment the space into parts corresponding to each BCC (named with a specific BCT). Following this reasoning, the differences between the color experiences of normal and dichromatic vision (see, Moreira et al., 2018) do not pose a problem in this interpretation, but forms part of the evidence suggesting that universal factors are not necessary to explain the origin and development of BCTs.

Despite assuming the relevance of the linguistic-cultural factors, and even assuming that they partially explain the differences in BCTs between languages (e.g., in their number), the universal evolution model (UE, Berlin and Kay, 1969; Kay and Maffi, 1999; Kay et al., 2009; Lindsey and Brown, 2014; Skelton et al., 2017) proposes that some universal factors are the main determinants of the key similarities found between very different languages (see Franklin, 2016, Figures 2.3 and 2.4). Among these universal factors are the physical regularities of the chromatic stimuli (Philipona and O’Regan, 2006) and/or the special characteristics of the opponent non-composite (unique) sensations (white vs. black, red vs. green, yellow vs. blue, see Forder et al., 2017; Lillo et al., 2018).

As stated before, in previous research (Moreira et al., 2014), we developed a very accurate model (the model B) to predict when Brown-Green dichromats would, and would not, be accurate in their use of BCTs. This model included three variables (L*_T_, *s´*, and *∆RG_res_*) specific to these types of observers, while related to some colorimetric variables developed by the CIE to describe normal trichromatic color vision. For example, L*_T_ (transformed lightness) is related to L* (CIE lightness variable) and has a similar interpretation. That is, despite the differences in the specific computed values for L*_T_ and L* for many stimuli (e.g., for red stimuli, L*_T_ values are lower than L* values for protanopes), both variables allow a very similar use to locate BCCs-BCTs: reds are always a set of dark, and not light, stimuli. The other two model B variables are used to delimitate experience types and magnitude (*s´* < 0, bluish colors; *s´* > 0, yellowish colors; and high *s´* values are saturated colors, etc.) and, therefore, are useful to define BCCs-BCTs. So, however different the color experiences of normal trichromats and Brown-Green dichromats are, these experiences still share important characteristics that make it seem possible that both types of observers use the same universal mechanisms for color-related tasks, as it has been previously demonstrated for color preference (Álvaro et al., 2015).

What cognitive strategies allow Brown-Green dichromats to approximate their BCT use to the normal trichromat use? That is, to use the same words for naming the same stimuli. We currently lack the information needed to answer this question, but we can speculate on several tentative answers. The first is related to categorical differentiation (Kay and McDaniel, 1978; see also, Lindsey and Brown, 2014), the development of a new category through the segregation of a region of the color space previously associated with one larger category. The Japanese language provides a nice example of the differentiation process. This language evolved from an 11 BCCs-BCTs language similar to the standard version of the American (Boynton and Olson, 1987; Lindsey and Brown, 2014) and British English (Sturges and Whitfield, 1995), Chinese (Lin et al., 2001) and Spanish (Lillo et al., 2007; Uusküla and Bimler, 2016) languages, to a 12 BCC-BCTs similar to some languages like Russian (Paramei, 2007), Italian (Paggetti et al., 2016), Greek (Androulaki et al., 2006) and a dialect of the Spanish language (Uruguayan, see Lillo et al., 2018). To be more specific, the category similar to the English blue identified as *ao* in Japanese at the end of the 20th century (Uchikawa and Boynton, 1987) became differentiated into two BCCs (Kuriki et al., 2017): one identified by the BCT *ao*, corresponding to dark blue; another identified as *mizu* applied to light blue. Importantly, the Spanish dialect spoken in Uruguay (Uruguayan) also contains two blues, “blue” (*azul*) and “sky” (*celeste*), similar to the new Japanese terms. Using a “boundary delimitation task,” Lillo et al. (2018) asked speakers of other Spanish dialects (Mexicans and Spaniards), lacking different BCTs for light and dark blues, to adjust the sky-blue boundary present in the Uruguayan dialect but absent in their own. This task requires the artificial establishment of a boundary between two hypothetical BCCs. The results showed that the new artificial boundary adjusted by both the Mexicans and Spaniards matched the actual Uruguayan boundary between blue and sky. Consequently, the boundary chosen for the hypothetical categories was not the result of arbitrary linguistic imposition but a universal light-dark differentiation.

The existence of a differentiation process in dichromatic children seems likely, since it may provide a way to delimitate BCCs progressively more similar to the BCCs of normal trichromats. It is possible, for example, that a differentiation based on *s´* (Moreira et al., 2014) could be related to a greater capacity to distinguish between blue and purple BCCs in protanopes. Since only the best blue exemplars have high *s´* values, protanopes would associate these with the use of the BCT “blue.” For a similar reason, low *s´* values would produce a preferential use of the BCT “purple.” Of course, this differentiation process cannot reproduce the normal trichromat use of “blue” and “purple” perfectly, because the distribution of *s´* values for blue and purple partially overlap, a fact that can be seen in the metacognition of protanopes (see less discriminable pairs in Tables 3 and 5).

The existence of compound BCCs is a hallmark of languages with fewer BCTs. These categories include colors that produce clear perceptual differences in the speakers of those languages. For example, it is common scientific jargon to speak of grue to refer to a compound category including exemplars of what for an English speaker would be greens and blues. In the case of Brown-Green dichromats, the emergence of the green BCC could also be the result of a learning process in which they use the same term to denominate colors that they can perceptually differentiate. For example, the BCT “green” in Brown-Green dichromats would include greens that can be confused with browns (low L*_T_ values, positive and medium *s*´ values), as well as greens that can be confused with grays (larger range of L*_T_ values, *s´* values near to zero, etc.). The logic behind the equilibrium of differentiation, and the creation of compound categories processes which allowed our adult participants a relatively good use of all the Castilian Spanish BCTs (denoting 11 BCCs, similar to the English ones) is still an enigma to be unveiled by future research.

In conclusion, although protanopes and deuteranopes experience a reduced gamut of colors, and even when looking at some stimuli they perceive different colors from those seen by normal trichromats, our research has shown that these observers have, besides a performance that exceeded expectations, an accurate metacognition of their use of BCTs, in the sense that their metacognition resembles their main difficulties differentiating BCTs used to name related colors on a daily basis. In this context, the Red-Green pair is not especially relevant (very probably due to red-green residual discrimination, see Moreira et al., 2014), therefore it seems adequate to replace the traditional denomination “Red-Green dichromats” with the new one “Brown-Green dichromats.” This last expression makes it easy to remember that BCTs are acquired and used similarly to normal trichromats within the context of related colors, and that undoubtedly; the Brown-Green pair is estimated to be the most problematic in the metacognition of these dichromatic observers, properly reflecting that the greatest empirical difficulties are related with this pair. Future research will have to determine which factors are involved, and what are their relative importance’s in the high level of discrimination between the BCTs observed in our research.

## Data Availability Statement

The raw data supporting the conclusions of this article will be made available by the authors, without undue reservation.

## Ethics Statement

The studies involving human participants were reviewed and approved by Hospital Clínico San Carlos Review Board of the Universidad Complutense de Madrid (Spain). Written informed consent to participate in this study was provided by the participants’ legal guardian/next of kin.

## Author Contributions

HM and JL designed the first experiment. HM, JL, and LÁ designed the second experiment. Data collection was performed by HM in the first experiment and by LÁ in the second experiment. HM, JL, and LÁ analyzed the data. JL drafted the manuscript. All authors reviewed, edited and approved the final version of the manuscript for submission.

### Conflict of Interest

The authors declare that the research was conducted in the absence of any commercial or financial relationships that could be construed as a potential conflict of interest.
